# Inflammatory Diseases and Growth: Effects on the GH–IGF Axis and on Growth Plate

**DOI:** 10.3390/ijms18091878

**Published:** 2017-08-31

**Authors:** Francesca Cirillo, Pietro Lazzeroni, Chiara Sartori, Maria Elisabeth Street

**Affiliations:** Division of Paediatric Endocrinology and Diabetology, Department of Obstetrics, Gynaecology and Paediatrics, Azienda AUSL–IRCCS, Viale Risorgimento, 80, 42123 Reggio Emilia, Italy; francesca.cirillo@ausl.re.it (F.C.); pietro.lazzeroni@ausl.re.it (P.L.); chiara.sartori@ausl.re.it (C.S.)

**Keywords:** bone, growth, inflammation, NF-κB, IGF system, GH–IGF axis, cystic fibrosis, inflammatory bowel disease, juvenile idiopathic arthritis, intrauterine growth restriction

## Abstract

This review briefly describes the most common chronic inflammatory diseases in childhood, such as cystic fibrosis (CF), inflammatory bowel diseases (IBDs), juvenile idiopathic arthritis (JIA), and intrauterine growth restriction (IUGR) that can be considered, as such, for the changes reported in the placenta and cord blood of these subjects. Changes in growth hormone (GH) secretion, GH resistance, and changes in the insulin-like growth factor (IGF) system are described mainly in relationship with the increase in nuclear factor-κB (NF-κB) and pro-inflammatory cytokines. Changes in the growth plate are also reported as well as a potential role for microRNAs (miRNAs) and thus epigenetic changes in chronic inflammation. Many mechanisms leading to growth failure are currently known; however, it is clear that further research in the field is still warranted.

## 1. Introduction

Most chronic inflammatory diseases in childhood are characterised by impaired growth. The mechanism underlying the pathophysiology of this process is not clearly understood yet, although it is a complex phenomenon which comprises of chronic inflammation itself, prolonged use of glucocorticoids, and suboptimal nutrition [1,2].

Longitudinal growth in humans is under the control of multiple factors, and its regulation starts during foetal life and continues throughout childhood, with varying influence of each factor at different stages. The main determinants of growth are prenatal and perinatal health, genetic potential, adequate nutrition, and endocrine interplay. In particular, hormones involved in this process are growth hormone (GH) and insulin-like growth factor (IGF) system, thyroid hormones, insulin, and sex steroids [3].

During the last decade, attention has been focused on epigenetics and its role in normal and pathological development, and a number of epigenetic mechanisms have been recognised as regulators of growth pattern [4].

Growth retardation may, therefore, be secondary to a dysfunction in multiple systems, such as disruption of the GH–IGF axis and IGF system, changes in the growth plate, epigenetic modifications and malnutrition.

The GH–IGF axis and IGF system in children with chronic inflammation may be altered by several mechanisms, such as GH/IGF-1 insufficiency, GH/IGF-1 resistance, down-regulation of GH/IGF receptors, disruption in downstream GH/IGF signalling pathways, or dysregulation of IGF binding proteins (IGFBPs) [1]. Pro-inflammatory cytokines play a crucial role in the development of these abnormalities. Many studies over the last decade clearly demonstrate huge interactions between pro-inflammatory cytokines and the IGF system.

The aim of this review is to give an overview of the main mechanisms underlying the onset of growth impairment in chronic inflammatory disease in childhood.

## 2. Overview of Most Frequent Chronic Inflammatory Diseases with Impaired Growth

Growth evaluations are among the most common referrals to paediatric endocrinologists, and many are owing to growth failure in chronic inflammatory diseases.

Cystic fibrosis (CF), inflammatory bowel disease (IBD), and juvenile idiopathic arthritis (JIA) are the most common chronic inflammatory conditions in childhood associated with growth impairment. Evidence in recent years has shown that intrauterine growth restriction (IUGR) also can be considered as a chronic inflammatory condition [5,6].

### 2.1. Cystic Fibrosis

CF is an autosomal recessive disorder caused by mutations in a gene that encodes for the cystic fibrosis transmembrane conductance regulator (CFTR) protein, an epithelial chloride channel that is widely expressed and is involved in the homeostasis of ions and other metabolites.

Chronic inflammation in the pathophysiology of CF has been extensively documented [7,8,9].

*CFTR* is widely expressed, and therefore, CF affects different organs and systems. Lung damage is mostly responsible for high morbidity and mortality, and is characterised by bronchiectasis, small airway obstruction, and progressive respiratory impairment.

However, epithelial cell dysfunction causes important comorbidities, such as malabsorption, biliary cirrhosis, and infertility [10]. The severity of CF varies greatly from person to person, regardless of age [11]. The endocrine system is also frequently involved in CF patients, with important consequences, including poor linear growth and diabetes [12]. The prevalence of short stature in patients with CF is approximately 20% [13].

Morison et al., reporting cross-sectional data from 31 CF Centres in the UK, indicated that during the first decade of life, height and weight in patients with CF are maintained at about 0.5 SDs below those of the general population, and fall away progressively after this age [14]. Delayed puberty may also be involved in the determinism of the reduction of linear growth in these patients. Several authors have shown how adolescents with CF present lower peak height velocity, with pubertal delay and a later pubertal growth spurt [15,16].

Short stature in CF may have an impact on disease severity because it is an independent predictor of mortality. This evidence may reflect a subgroup of CF patients with poorer nutrition, or chronic inflammation and ongoing pulmonary exacerbation [17].

Fat and micronutrient malabsorption may contribute to poor growth. However, other factors involved in the determinism of growth failure in CF patients include chronic inflammation, chronic infection and treatment with inhaled and systemic glucocorticoid medications.

There is now sufficient evidence to suggest that poor growth in CF is already seen in the neonatal period, and that the CF genotype delF508 plays a contributing role [1].

### 2.2. Inflammatory Bowel Diseases

IBDs are conditions characterised by chronic or recurring immune response and inflammation of the gastrointestinal tract. The two most common IBDs are ulcerative colitis (UC) and Crohn’s disease (CD).

IBDs are more common in developed countries. There is north-to-south variation, and they are more common in urban communities compared with rural areas. These observations suggest that urbanisation is a potential contributing factor.

According to Centres for Disease Control and Prevention (CDC) and the USA National Health Protection Agency, although the incidence and prevalence of UC and CD are beginning to stabilise in high-incidence areas, such as northern Europe and North America, they continue to rise in low-incidence areas, such as southern Europe, Asia, and much of the developing world [18].

These conditions affect as many as 1.4 million persons in the United States, and 2.2 million persons in Europe [19].

Pathophysiology of IBDs is complex, and most authors agree on the fact that these conditions result from interactions between environmental factors, genetic predisposition, and immune response.

Intestinal epithelial damage with infiltration of a large number of cells into the lamina propria, such as T and B lymphocytes, macrophages, dentritic cells, and neutrophils, is a constant occurrence along with IBDs [2,4,12]. Moreover, evidence suggests a defect of immune response regulation in these conditions, with active secretion of a large number of cytokines with both pro-inflammatory and anti-inflammatory action, including TNF, IFN-γ, IL-6, IL-12, IL-21, IL-23, IL-17, integrin, IL-10, TGFβ, and IL-35 [2,11]. The imbalance in the regulation and secretion of these cytokines plays a crucial role in initiating and sustaining intestinal inflammation and tissue injury.

Both these diseases are responsible for severe gastrointestinal symptoms, such as abdominal pain, diarrhoea, rectal bleeding, nausea/vomiting or constipation, with a potential huge reduction in quality of life.

However, the clinical presentation of IBDs in children and adolescents can be variable, and up to 22% of children may present with extra-intestinal manifestations as the only predominant initial feature. Main extra-intestinal manifestations of IBDs are growth failure, anaemia, erythema nodosum, pyoderma gangrenosum, arthritis, perianal disease, osteopenia, osteoporosis, primary sclerosing cholangitis, autoimmune hepatitis, episcleritis, uveitis, and pancreatitis [20]. Impaired linear growth is a frequent extra-intestinal complication of IBDs in children and adolescents. Up to 19–31% of children with CD and UC present with this sign as an initial feature [21,22,23]. Moreover, early growth delay has been associated with permanent growth retardation in 17% of patients [24].

### 2.3. Juvenile Idiopathic Arthritis

JIA is a heterogeneous group of diseases characterised by arthritis of unknown origin, with onset before age of 16 years. JIA is a common childhood rheumatic disease. The highest frequency of this condition occurs in children aged 1–3 years, and its incidence is around 20–30/100,000 below the age of 16 years [25].

JIA classification comprises different subtypes (systemic arthritis, polyarthritis, oligoarthritis, enthesitis-related arthritis, psoriatic arthritis, and undifferentiated arthritis) which are characterised by distinct clinical features and varying spectrums of disease severity [26].

Pathogenesis of JIA involves the humoral and cell-mediated immune system. T lymphocytes play a pivotal role, releasing pro-inflammatory cytokines and promoting type-1 helper T lymphocyte response. An abnormal interplay between type 1 and type 2 T helper cells has been shown [27].

Growth impairment is a well-known long-term complication in patients with JIA. Children with systemic or polyarticular JIA (about 30–40% of all JIA cases) present a higher incidence of growth disturbances among all JIA patients [28].

Children with arthritis suffer from a variety of growth disorders, ranging from general growth retardation to local acceleration of growth in the affected limb [29,30]. Leg-length discrepancy is common, as unilateral knee arthritis may result in overgrowth of the distal femur, caused by increased blood supply to the inflamed joint with consequent accelerated growth of the ossification centres.

Puberty may also be involved with retardation in the appearance of secondary sex characteristics [31]. Risk factors for impaired linear growth are represented by extended periods of active disease, and are exacerbated by long-term use of systemic steroids.

Moreover, an elevated erythrocyte sedimentation rate value seems to be a good predictor of risk for growth retardation [32].

### 2.4. Intrauterine Growth Restriction

IUGR is defined as the failure of a foetus to attain its expected foetal growth at any gestational age. It affects approximately 7–15% of pregnancies with an estimated prevalence of 8% in the general population [33].

The incidence of IUGR varies among countries, populations, and races, and increases with decreasing gestational age. Around 14 to 20 million infants have been affected with IUGR in developing countries annually. A large number of IUGR infants are seen in the also Asian, African and Latin American continents [34]. IUGR represents therefore one of the most important causes of perinatal mortality and morbidity worldwide.

The main determinants of foetal growth are genetic heritage of the foetus, the integrity of the materno–placento–foetal unit, adequate nutrient and oxygen supply, and the right hormonal milieu. Impaired foetal growth may be due to inadequacy of any one of these parameters.

Risk factors for the development of IUGR are, therefore, extremely variable, and may include maternal medical and social conditions, foetal abnormalities, and placental dysfunction.

IUGR puts the foetus and neonate at higher risk for perinatal mortality and morbidity [35], and it is also considered a risk factor for the future development of insulin resistance and type 2 diabetes [36].

These conditions are related by association with high serum concentrations of pro-inflammatory cytokines, such as interleukin (IL)-6 and tumour necrosis factor α (TNF-α), and low adiponectin concentrations [36,37,38,39,40,41,42].

Interestingly, IL-6 concentrations in placental lysates from IUGR pregnancies correlate with birth length, birth weight, and head circumference [36].

The increased IL-6 concentrations confirm the hypothesis that IUGR shares a common pathophysiology with other conditions characterised by chronic inflammation.

Within the first two years of life, especially in developed countries, the majority of IUGR subjects present partial or complete catch-up growth. However, approximately 13% of these subjects do not present a catch-up growth, and remain short after two years of life [43].

## 3. Interactions between Pro-Inflammatory Cytokines and GH–IGF Axis, IGF System, and Bone

As stated above, the mechanisms underlying the pathophysiology of growth impairment in chronic inflammatory diseases in childhood is a complex phenomenon in which chronic inflammation plays a central role [1,2].

### 3.1. GH–IGF Axis and IGF System

GH is a 191 amino acid protein that promotes growth by increasing cell size and cell number, and by promoting differentiation of bone and muscle cells [44]. Deficiency in either GH or the GH receptor causes severe postnatal growth retardation and subsequent dwarfism, both in humans and mice [45,46]. IGF-1 is expressed by most cells throughout development, and represents an essential factor for cell growth, intrauterine development, and postnatal growth [47,48,49,50]. IGF-1 deficiency in humans and mice causes severe intrauterine and postnatal growth retardation, and is associated with perinatal lethality, and developmental defects in the bone, muscle, and central nervous and reproductive systems. The effects of GH on postnatal body growth have been extensively described [51,52]. Briefly, GH acts on a major target organ, the liver, to stimulate the synthesis and secretion of IGF-1, which reaches its skeletal targets as a true endocrine reagent (the somatomedin hypothesis) [53], but it can stimulate longitudinal bone growth directly, also, by local production of IGF-1 (modified somatomedin hypothesis) [54,55,56,57,58,59]. Both IGF-1 and GH are necessary for postnatal growth [60]. IGF-1 regulates with a negative feedback loop mode of action on GH secretion at the pituitary level. In cartilage cells, IGF-1 has GH-independent stimulating effects, but its effects are optimised by a synergistic action with GH itself [61].

GH binds to two GH receptors (GHRs), causing a dimerisation process that activates the GHR-associated Janus kinase (JAK)2 tyrosine kinase, and tyrosine phosphorylation of both JAK2 and GHR. These events activate a series of other signalling molecules, such as mitogen-activated protein kinases (MAPKs), insulin receptor substrates, phosphatidylinositol-3-phosphate kinase, diacylglycerol, protein kinase C, intracellular calcium, and signal transducer and activator of transcription (STAT) factors. These signalling molecules lead to changes in enzymatic activity, transport function, and gene expression that determine final changes in growth and metabolism. The GH binding protein is proteolysed from the cell surface receptor, and regulates GH bioavailability, and can be used as a marker of receptor number and function [52].

The IGF system is of upmost importance for somatic growth in vertebrates. Both IGF-1 and IGF-2 signal through the IGF type 1 receptor (IGF-1R). IGF-1R regulates proliferation, differentiation, and apoptosis in many tissues and cell types. IGF-1R is a transmembrane tyrosine kinase receptor. Both ligands and the IGF-1R are similar to insulin and the insulin receptor [62,63]. Six IGF binding proteins (IGFBPs) and the mannose-6-phosphate receptor (type 2 IGF receptor) are the main regulators of IGF-1 and IGF-2 bioavailability [64,65,66].

### 3.2. Changes in the GH–IGF Axis and IGF System and Interactions with Pro-Inflammatory Cytokines

**Figure 1:**
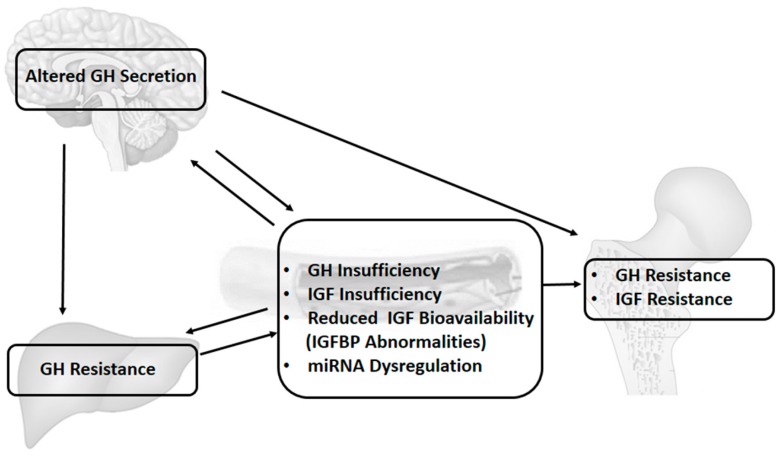
Overview of actions of pro-inflammatory cytokines on the growth hormone (GH)–insulin-like growth factor (IGF) axis and IGF system. Pro-inflammatory cytokines determine a dysregulation in GH–IGF axis and IGF system, both at a central and peripheral level. In the brain, inflammation determines a dysregulation of GH secretion from the pituitary gland. In the liver, a pro-inflammatory cytokine milieu results in a down-regulation of GH receptors and in impaired downstream signalling, with subsequent GH resistance and IGF-1 insufficiency. GH and IGF-1 resistance are present in the growth plate. Abnormalities in IGF binding proteins (IGFBPs), with reduction in IGF bioavailability, are a constant feature. MicroRNAs (miRNAs) targeting genes within the GH–IGF axis and IGF system are dysregulated, and potential key mediators of these processes.

The GH–IGF axis may be altered by several mechanisms, and growth failure in children with chronic inflammatory conditions may be secondary to GH/IGF-1 insufficiency, GH/IGF-1 resistance, down-regulation of GH/IGF receptors, disruption in downstream GH/IGF signalling pathways, dysregulation of IGFBPs and thus of IGF bioavailability, and finally, gene regulation that is modified by changes in the microRNA system, as well as other potential epigenetic mechanisms (Figure 1).

Pro-inflammatory cytokines play a crucial role in the development of these abnormalities. Many studies over the last decade clearly demonstrate huge interactions between pro-inflammatory cytokines and the IGF system.

For example, studies in transgenic mice showed how increased IL-6 serum levels were associated with low IGF-1 serum levels and growth delay [67]. Street et al. showed also a relationship between inflammatory status and the IGF system, with a consequent effect of these interactions on longitudinal growth [36,68].

IL-6 may antagonise GH actions through disruption of JAK/STAT signalling. Recent evidence suggests a role for suppressor of cytokine signalling (SOCS) family proteins in these processes. Pro-inflammatory cytokines stimulate SOCS proteins with a consequent reduction in JAK2 and STAT activation [69,70,71]. Similarly, an abnormal expression of STAT5 and STAT3, due to the action of another crucial pro-inflammatory cytokine, IL-1β, can disrupt GH signalling [72].

The IGF-1 signalling pathway may also be altered in chronic inflammatory conditions. Several authors showed how TNF-α, IL-6, and IL-1β dysregulate IGF-1 intracellular mediators MAPK/extracellular signal-regulated kinases (ERKs), and phosphoinositide 3-kinase (PI3K), in chondrocytes [73,74,75]. Inflammation is sustained by the activation of several immune cell types which secrete soluble cytokines, as chemokines, interferons, and ILs, activating bone resorption and inhibiting bone growth/formation processes at local and systemic levels [76].

These messenger molecules influence differentiation and activity of the main skeletal cell types: osteoblasts, osteoclasts, and chondrocytes [77,78].

Experimental and clinical evidence suggests that the nuclear factor-κB (NF-κB) pathway plays a role in IGF1-GH signalling [79,80], and exerts a regulatory role in bone growth and development [81,82,83]. NF-κB is a family of transcription factors which can form homo- and heterodimers, including the five members, p50 (NF-κB1), p52 (NF-κB2), p65 (RelA), RelB, and c-Rel. All of these proteins are structurally homologous and share functional domains: N-terminal Rel homology domain (RHD), responsible for dimerisation as well as DNA-binding, and a transactivation domain (TA) relevant for the transcriptional activity. Moreover, they present a nuclear localisation signal (NLS) that promotes translocation into the nucleus from the cytoplasm after stimulation [84].

In resting cells, NF-κB dimers are retained in the cytoplasm, where they are covalently bound to the inhibitor of NF-κB (IκB) proteins, which mask their NLSs.

The main and most well studied NF-κB activation pathways are canonical, and alternative pathways differ mainly in composition of downstream dimeric effectors.

NF-κB pathways are activated by many extracellular signals, e.g., pro-inflammatory cytokines, hormones, growth factors, viral proteins, lipopolysaccharide (LPS), and RANKL.

In the activated classic pathway, the IκB kinase (IKK) induces the ubiquitination and subsequent degradation of IκB. Subsequently, NF-κB, predominantly p50/p65, translocates into the nucleus, where it regulates genes involved in cell proliferation, differentiation, and death [85].

In the alternative pathway, the key point is represented by the partial proteolysis of p100 or p105 precursors of p52 and p50, respectively, which allow the activation and subsequent translocation of p52/RelB or p50/p50, predominantly [86,87].

### 3.3. Inflammation and miRNAs

Epigenetics, defined as “the inheritance of variation (-genetics) above and beyond (epi-) changes in the DNA sequence” [88], refers to inheritable changes of gene function, which do not imply a change in the DNA sequence [89].

MicroRNAs (miRNAs) are a recent chapter in the study of epigenetic regulation. They are endogenous small non-coding RNAs, approximately 22 nucleotides long that act as post transcriptional regulators [90]. Mature miRNAs hybridise to partially complementary binding sites that are typically localised in the 3′ untranslated regions (3′UTR) of target mRNAs [91].

Upon binding, miRNA can cause the degradation of the mRNA target if the complementarity between them is perfect; when their complementarity is only partial, the target’s translational repression takes place [92,93]. In these ways, each single miRNA regulates several hundreds of transcripts, and each mRNA can be regulated by many miRNAs [94,95].

In turn, miRNAs themselves are targets of transcription factors and molecular signals, thus explaining the complexity of the regulatory network existing on and controlled by miRNAs [96,97].

The complexity of miRNA action, and their strict regulation, provides the evidence that miRNAs are crucial in various physiological and pathological processes; in fact, they are involved in inflammation, apoptosis, differentiation, and proliferation [98].

Moreover, miRNAs have a role in the antigen-presenting capacity and costimulation activity of macrophages and dendritic cells [99,100], and they are also essential in the development and actions of B and T cells [84,101,102].

In particular, under inflammatory stimuli, miRNAs act as post-transcriptional regulators of genes involved in the adaptive and innate immune response; for example, they control the expression of mediators involved in the Toll like receptor (TLR) signalling pathways, which all culminate in the activation of NF-κB [103,104,105].

During many biological processes, like inflammation or innate and adaptive immunity, NF-κB regulates the transcription of miRNAs through the induction of genes codifying for regulatory proteins [106].

NF-κB targets miRNA sequences, including miR-9, miR-21, miR-143, miR-146, and miR-224 [103,107,108,109,110,111], which are involved in feedback mechanisms regulating the transcription of NF-κB itself.

miRNAs have the potential to influence the expression of the proteins involved in the regulation of GH–IGF axis. Their deregulation has been well documented in association with chronic paediatric diseases in relationship to the inflammatory state, as in CF [112,113,114,115], IBD [116,117,118,119,120,121,122], JIA [123,124], and IUGR [125,126].

### 3.4. Bone Growth and Inflammation

Bone has a lot of functions, such as structural support, calcium reserve, and many others. During life, bone undergoes modelling and remodelling, which is due to the action of two major cell types: osteoblasts and osteoclasts. Modelling is an adaptive process by which bones answer to external influences to adjust the skeleton to events occurring during life [127].

Bone renewal occurs physiologically by bone remodelling. This process is necessary for maintaining bone strength and mineral homeostasis [127].

Longitudinal growth occurs only during childhood. It is only in this period that bone formation can occur independently of bone resorption [128]. The process of longitudinal growth occurs in growth plates. An alteration between bone formation and bone resorption occurs in chronic inflammatory conditions. Inflammation is a primary cause of bone loss. This bone loss is both local and systemic, and is associated with an enhancement of bone resorption or an inhibition of bone formation.

Most of chronic paediatric inflammatory diseases are associated with a catabolic state that reduces bone formation [128].

The epiphyseal growth plate is the final target organ of the above described growth-regulating mechanisms. The epiphyseal growth plates are located in the proximal and distal parts of the long bones and have a definite cellular organisation according to stage of maturation, with germinative, proliferative, hypertrophic, and degenerative cell layers. The germinative cell layer consists of stem cells or progenitor cells, which rarely divide. During the process of longitudinal bone growth, stem cells enter the proliferative cell layer and begin to divide frequently, forming continuous cell columns parallel to the longitudinal axis of the bone. Subsequently, these cells stop dividing, mature, and become part of the hypertrophic cell layer [129]. Finally, calcification occurs as cartilaginous matrix is transformed into bone matrix. Longitudinal bone growth is due to the recruitment of new progenitor cells from the stem cell layer that undergo divisions in the proliferative layer, and then increase in size in the hypertrophic layer. The growth plate is a constantly renewing tissue that pushes the epiphysis further and further away from the centre of the long bone [130].

Linear growth occurs during development and the childhood years until epiphyseal fusion occurs. This is due to endochondral ossification, and is regulated by systemic hormones and paracrine or autocrine factors. Childhood growth requires GH, IGF-1, glucocorticoids, and thyroid hormone to be present and normally active. Sex steroids are then additionally necessary for the pubertal growth spurt and epiphyseal fusion [131]. Furthermore, during linear growth, GH, IGF-1, glucocorticoids, and thyroid hormone interact at the level of the hypothalamus and pituitary. However, recent evidence suggests that these hormones also act directly on peripheral target tissues, such as liver and growth plate [132].

GH action has both direct and indirect effects on the growth plate. GH acts indirectly, stimulating the production of IGF-1 that promotes chondrocyte hypertrophy, which in turn exerts its effects on the growth plate. The direct effect of GH on the growth plate stimulates chondrocyte proliferation [133].

The balance between bone degradation and bone building is critical for the physiological bone homeostasis. NF-κB and cytokines controlled by this factor may perturb this equilibrium in paediatric chronic inflammatory diseases [134,135,136].

NF-κB activation is a relevant component for osteoclast development, differentiation, and survival, cooperating with other pro-inflammatory cytokines. Loss of NF-κB signalling prevents osteoclastogenesis [82]. NF-κB knockout mice showed severe osteopetrosis [137].

Furthermore, NF-κB inhibits osteoblast differentiation by blocking transcription factors induced by several extracellular signals, including bone morphogenic proteins (BMPs), fibroblast growth factor (FGF), transforming growth factor β (TGF-β), and transducers of GH–IGF-1 axis. The inhibition of bone formation by NF-κB is well documented by in vivo and in vitro experiments [138,139].

**Figure 2:**
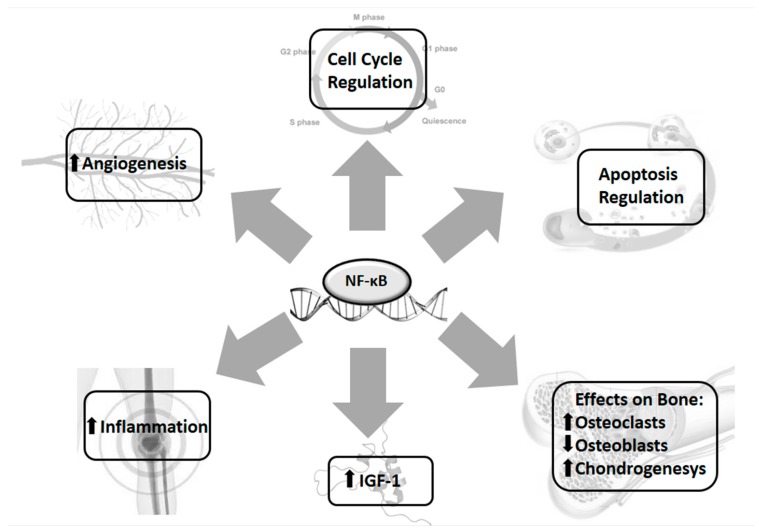
NF-κB as a central transcriptional factor in the inflammatory process. Nuclear factor-κB (NF-κB) is a transcriptional factor with a crucial role in the control of mechanisms mediated by inflammation. It orchestrates several processes, such as angiogenesis, apoptosis, and cell cycle regulation, and is important as a regulator of bone remodelling, enhancing osteoclast and reducing osteoblast activity. Moreover, it influences IGF-1 secretion, and it represents one of the factors linking inflammation with growth.

NF-κB is also involved in the regulation of growth plate chondrogenesis by IGF-1, which promotes bone longitudinal growth during childhood and foetal development by stimulating chondrocytes proliferation and preventing apoptosis [140,141]. Various stimuli, such as TNF-α, LPS, and hypoxia, increase the expression of IGF-1, vascular endothelial growth factor (VEGF), and FGF-2 by an NF-κB dependent mechanism [142] (Figure 2).

Another system involved in growth failure in the chronic inflammation state is represented by the adrenal axis. Circulating levels of endogenous glucocorticoids (GC) increase during inflammation, leading to growth suppression. Steroids act both at systemic and local level, as well as the GH system. Localised GC directly inhibits chondrocyte proliferation and bone mineralisation, as well as increases apoptosis. By systemic action, GC inhibits GH secretion, with a consequent reduction of IGF-1 production and activity [143].

Glucocorticoids are frequently used for the treatment for many inflammatory diseases. Initially, steroids could have beneficial effects on bone growth, suppressing inflammation, but long-term therapy frequently has adverse effects on bone [144]. High levels of circulating glucocorticoids disrupt bone remodelling, disrupting the balance between formation and resorption. Glucocorticoids act directly on osteoblasts, decreasing their proliferation and the consequent production of specific proteins as osteocalcin, a bone specific alkaline phosphatase [145,146]. Enhanced adverse effects of glucocorticoids are partly dependent on the underlying illness [147,148].

## 4. Specific Changes in the GH–IGF Axis, IGF System, and Growth Plate in Individual Inflammatory Conditions

In CF patients, studies of GH secretion are limited. Arginine and clonidine GH stimulation tests, performed in a group of adolescents with CF, revealed how approximately 50% of these patients have peak GH levels <6 µg/L, and IGF-1 levels of −0.5 SDs, suggesting the potential co-existence of GH insufficiency and GH resistance [149].

A significant positive correlation was indeed found between insulin secretion and height growth velocity and serum IGFBP-3 levels [149]. According to data published by our group, CF patients have higher serum concentrations of IL-1β, IL-6, TNF-α, and IGFBP-2. Conversely, serum concentrations of IGF-1 and IGF-2 are significantly lower. IGFBP-3 serum concentrations are similar, with comparable IGF-1/IGFBP-3, and decreased IGF-1/IGFBP-2 and IGF-2/IGFBP-2 molar ratios.

Statistical analyses revealed a significant positive correlation between IGFBP-2 and IL-6 and a negative correlation between IGFBP-2 and IGFBP-3, suggesting that inflammation is an important modulator of the IGF-IGFBP system with an overall reduction in IGF bioactivity in CF [68].

Moreover, circulating levels of TNF-α, IL-6, insulin, and the IGF system were found to be related to linear growth in children with CF [36].

IGF-1 concentrations in patients with CF are significantly lower than those in a healthy control population. IGF-1 reduction in these patients may reflect their catabolic state and play a part in their abnormal growth pattern [150,151].

Animal models have shown that chondrocytes express functional CFTR [152], and cartilage abnormalities in tracheal ring structure have been reported in CF, both in humans and in animal models [153,154]. Both CFTR loss of function and local and systemic inflammation are hypothesised to be responsible for these changes, however, it is yet unclear whether cartilage abnormalities involve growth plate chondrocytes also, as these data are missing to date in the literature.

The precise mechanisms underlying growth failure in IBDs are not well known. The relative roles of impaired nutrition and active inflammation in disturbing GH–IGF axis remain controversial. Nutritional status regulates the IGF system, with both caloric and protein restriction resulting in low serum IGF-1 and IGFBP-3 levels. Serum IGFBP-2 regulation is more dependent on protein intake [155]. However, it was demonstrated that linear growth impairment occurs, independent of undernutrition, as a direct result of the inflammatory process. According to Ballinger and his group, in a rat experimental model of colitis, approximately 30–40% of linear growth impairment was directly related to inflammation [156]. These same authors suggested a normal stimulated and spontaneous GH production in children with CD, and growth failure with a low IGF-1 plasma concentration, conditions compatible with GH resistance [157]. Moreover, we previously described low IGF-1 and high IGFBP-2 levels related to disease activity and anatomical distribution, consistent with active inflammation modifying the IGF–IGFBP system.

Intestinal inflammation is well known to have a negative impact on bone health. Scientific evidence has confirmed a reduction in bone mineral density in patients with IBD, and changes in the growth plate cartilage, in addition. In detail, induction of moderate intestinal inflammation in young male mice has been reported to reduce growth plate thickness and induce a hypertrophic response in chondrocyte matrix [158]. Specific data in humans are still missing in the literature.

Impairment of GH–IGF axis in JIA may be due to several mechanisms, ranging from GH secretion abnormalities to GH resistance or increased IGF-1 clearance [159]. Templ et al. showed that GH response to GHRH was reduced in patients with newly diagnosed rheumatoid arthritis, compared to healthy controls [160], and others showed how an inflammatory cytokine milieu caused impairment of target cell sensitivity to GH. GH resistance in JIA seems to be a consequence of reduced GHR expression, or changes in intracellular signalling (deactivation of GHR/JAK2 complex, inhibition of JAK/STAT signalling by SOCS) [161,162].

GHR mRNA expression has been reported to be significantly reduced in mononuclear cells of JIA patients at the onset of the disease, with a restoration of GHR expression after two years of treatment [163]. However, these data remain controversial, as other authors did not report changes in *GHR* gene expression in a rat experimental arthritis model [164]. Granado et al. reported a downregulation in liver *IGF-1* gene expression in another experimental arthritis model [165], while others showed that circulating IGFBPs are increased in arthritis, resulting in reduction in IGF-1 bioavailability [166]. Furthermore, acid labile subunit (ALS) seems to be decreased in children with JIA [167].

JIA is characterised by significant changes in the articular microenvironment. Immune cell proliferation causes localised hypoxia and a reduction of pH. As a consequence, osteoblast function is impaired and bone mineralisation reduced [168].

In addition, negative direct effects on growth plate mediated by inflammatory cytokines have been described, in particular, TNF-α and IL-1β inhibit chondrocyte proliferation and function [169]. Moreover, an additive negative effect of IL-1β and TNF-α on bone growth has been described [74].

The IGF system is central to foetal growth, and relationships between cytokines and the IGF system have been shown in the placenta and cord serum of IUGR foetuses. Changes are reported also in children born with IUGR in the following years.

During foetal life, an adequate nutrient supply mainly determines foetal growth, while the intervening growth factors have rather a paracrine or autocrine role to play at the local level, to assure the availability of a nutrient supply, and to promote functional differentiation of the different tissues and organs. These growth factors are mainly insulin, IGF-1, and IGF-2. The IGF axis plays a critical role also in placental development and function. IGF signalling (specifically IGF-2, IGFBP-1, and IGF-1R) plays a critical role in trophoblast invasion and increased utero–placental blood flow during implantation, while imbalances or abnormalities in this signalling lead to adverse pregnancy outcomes, and have been associated with IUGR [170]. Defects in IGF signalling, may lead to impaired foetal growth [171].

Cord serum IGF-2 was described to be lower in IUGR compared with adequate for gestational age newborns, whereas IGFBP-2 was higher [172]. IUGR neonates present higher placental concentrations of IGF-2, IGFBP-1, IGFBP-2, and IL-6 [6].

During childhood, GH status in IUGR is not completely clear. GH responses to provocative stimulation tests and serum levels of IGF-1 and IGFBP-3 are reported to be normal in the majority of patients with normal IGF bioavailability [173,174]. However, in some studies, high pulse frequency and attenuated pulse amplitude related to GH secretion, have been reported [173,174]. These authors described reduced IGF-1, IGF-2, and IGFBP-3 serum concentrations, and reduced spontaneous GH secretion also [175,176].

Traditionally, a delay in bone maturation has been observed in patients with IUGR [177,178]. However, to date, there are no data regarding specific structural abnormalities in growth plate in these patients.

## 5. Conclusions

Inflammation is a clear cause of growth impairment. Mechanisms related to GH secretion and resistance, changes in the IGF system, and some changes in the growth plate, have been quite extensively studied, but are not fully elucidated yet. Furthermore, new mechanisms are arising, such as changes reported in the miRNA system that need to be addressed in the near future, in order to improve treatment of inflammation and growth.
